# Therapeutic Strategies For Tay-Sachs Disease

**DOI:** 10.3389/fphar.2022.906647

**Published:** 2022-07-05

**Authors:** Jaqueline A. Picache, Wei Zheng, Catherine Z. Chen

**Affiliations:** National Center for Advancing Translational Sciences, National Institutes of Health, Bethesda, MD, United States

**Keywords:** drug development, enzymatic assay, phenotypic screen, high throughput screen (HTS), mass spectrometry, tay-sachs disease (TSD)

## Abstract

Tay-Sachs disease (TSD) is an autosomal recessive disease that features progressive neurodegenerative presentations. It affects one in 100,000 live births. Currently, there is no approved therapy or cure. This review summarizes multiple drug development strategies for TSD, including enzyme replacement therapy, pharmaceutical chaperone therapy, substrate reduction therapy, gene therapy, and hematopoietic stem cell replacement therapy. *In vitro* and *in vivo* systems are described to assess the efficacy of the aforementioned therapeutic strategies. Furthermore, we discuss using MALDI mass spectrometry to perform a high throughput screen of compound libraries. This enables discovery of compounds that reduce GM2 and can lead to further development of a TSD therapy.

## Tay-Sachs Disease Overview

### Disease Etiology

**FIGURE 1:**
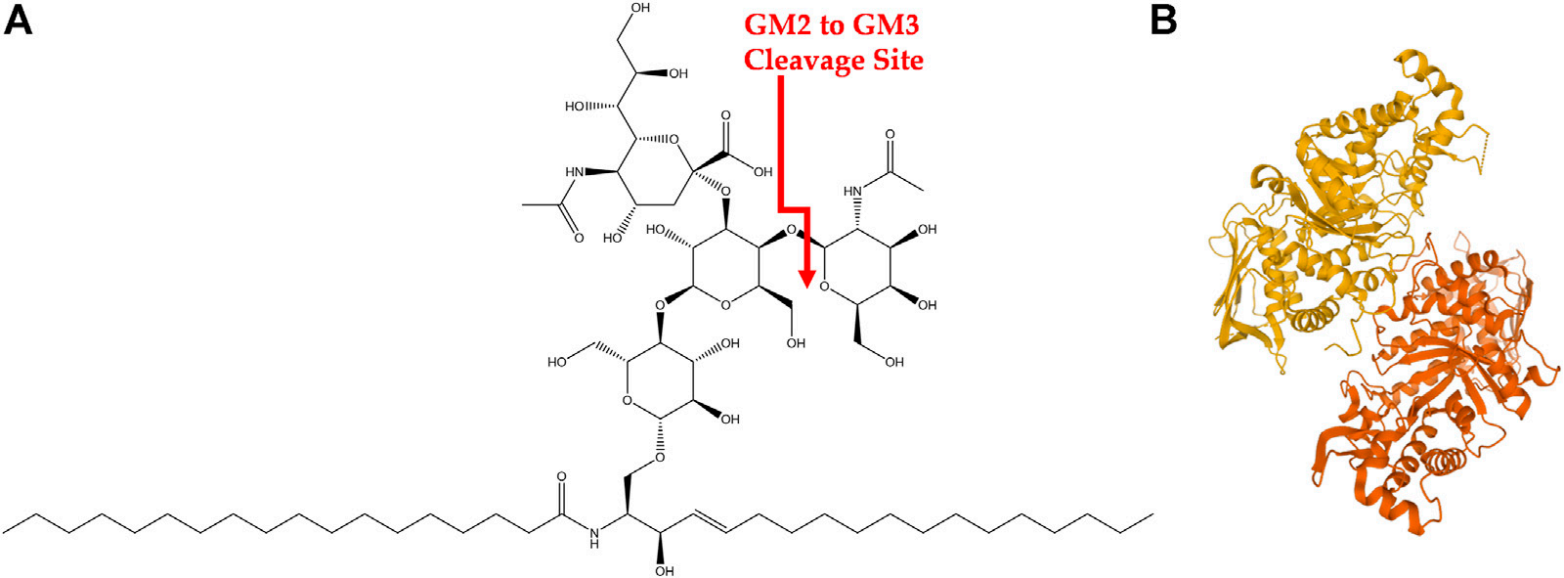
GM2 Ganglioside and HexA **(A)** Structure of GM2 ganglioside. Cleavage site of GM2 into GM3 *via* HexA is shown in red. **(B)** HexA isoenzyme with alpha subunit (orange) and beta subunit (yellow).

GM2-gangliosidoses are a group of three lysosomal storage disorders (LSDs) that result from a deficiency in one of the lysosomal enzymes β-hexosaminidases (Hex A, B, or S) or the GM2 activator protein (GM2A). This deficiency prevents the degradation of GM2 ganglioside (GM2), shown in Figure 1A, into GM3 ganglioside and causes a cytotoxic accumulation of GM2 (Leal et al., 2020). Gangliosides are glycosphingolipids that are essential in the central nervous system with multiple biological functions and are located within the cell membrane of neurons (Leal et al., 2020). Specifically, about five percent of glycosphingolipids within the central nervous system are GM2 gangliosides (Leal et al., 2020). Three LSDs associated with GM2-gangliosidosis are Tay-Sachs disease (TSD, OMIM #272800), Sandhoff’s disease (SD, OMIM #268800), and GM2 activator protein deficiency (also called AB variant, OMIM #272750), and are associated with pathogenic variants in *HEXA*, *HEXB*, and *GM2A*, respectively. This review focuses on TSD.

TSD is a congenital, autosomal recessive, neurodegenerative disease caused by mutations in the *hexosaminidase A* (*HEXA*) gene. It has an incidence rate of about one in 100,000 live births with a carrier frequency of about one in 250 (Lew et al., 2015; Solovyeva et al., 2018). However, the incidence rate is higher in certain founder groups such as the Ashkenazi Jewish population and those of Eastern European descent among others (Frisch et al., 2004). There are over 130 known *HEXA* mutations that lead to alterations in transcription, translation, protein folding and dimerization, as well as catalytic function (Hitoshi Sakuraba, 2006; Mistri et al., 2012; Solovyeva et al., 2018). Briefly, the *HEXA* gene encodes for the alpha subunit of the Hex isoenzyme HexA, shown in Figure 1B, which is comprised of an alpha and beta subunit. The alpha subunit of HexA is necessary to hydrolyze the N-acetylgalactosamine residue of GM2 during the degradation process (Leal et al., 2020). Various mutations in HexA result in a wide gradient of reduced enzymatic function and thus a wide spectrum in clinical severity of TSD. However, only 10–15% of residual enzymatic activity is necessary to avoid GM2 gangliosidosis (Osher et al., 2015; Solovyeva et al., 2018). Specific clinical manifestations of TSD are discussed in the next section.

### Disease Pathogenesis

There is a wide spectrum of clinical signs and symptoms in TSD patients. Generally, TSD patients have neurological signs and symptoms and do not present systemic issues (Leal et al., 2020). This is contrary to SD where patients often present organomegalies in addition to neurological degenerative symptoms (Leal et al., 2020). Diagnostic criteria for TSD include assessment of neurological signs and symptoms—specifically utilizing CT and MRI scans to measure cerebral white matter and basal ganglia hypodensity (Ramani and Sankaran, 2022). Once neurological signs and symptoms are assessed, patients may undergo HexA enzymatic function blood tests and genetic testing for known pathogenic variants to confirm diagnosis. Prenatal genetic testing is available for patients in populations with higher carrier frequencies (Ramani and Sankaran, 2022).

The TSD clinical spectrum is delineated into three main types of TSD based on symptom severity and age of onset of disease. The first type of TSD is the acute infantile form which is the most prevalent type. Acute infantile TSD signs and symptoms begin to manifest around 6 months of age and progress until lethality around age four (Bley et al., 2011). These patients have little to no (<0.5%) functional HexA and therefore GM2 accumulation occurs rapidly (Solovyeva et al., 2018). Common signs and symptoms of acute infantile TSD include mental and motor developmental delays such as hypotonia, inability to hold one’s head up or sit unsupported, eye movement abnormalities, development of a cherry red spot in the retina, seizures, and hypomyelination (Bley et al., 2011; Jarnes Utz et al., 2017; Solovyeva et al., 2018; Leal et al., 2020).

The next type of TSD is the juvenile subacute form. This type has an age of onset of three to 5 years old and is usually lethal by age 15 (Maegawa et al., 2006; Solovyeva et al., 2018). These patients have minimal residual HexA function though the diagnostic percentage of enzymatic function remains to be studied. The most common signs and symptoms, observed in 88% of juvenile subacute patients, are limb muscle weakness and ataxic gait (Maegawa et al., 2006). Other signs and symptoms include dysarthria, dysphagia, hypotension, intellectual disability, and cerebellar atrophy (Maegawa et al., 2006; Solovyeva et al., 2018; Leal et al., 2020). Overall, juvenile subacute TSD is less aggressive than its infantile counterpart. However, it has a wider range of signs and symptoms which can make diagnosis more difficult.

The third type of TSD is the adult chronic form characterized by its later onset of symptoms. Patients usually demonstrate signs and symptoms in their late adolescence/early adulthood. Some patients may not show symptoms until they are in their late 20 s or early 30 s (Sandhoff and Christomanou, 1979; Solovyeva et al., 2018). Late-onset Tay-Sachs disease (LOTS) presents with a wide variety of signs and symptoms, which could be misdiagnosed as other neuromuscular diseases or even psychiatric disorders (MacQueen et al., 1998; Bicchi et al., 2015). One case study indicated that adult chronic TSD patients may have 15% residual HexA activity (Barritt et al., 2017). Signs and symptoms include dysphagia, muscle atrophy, cerebellar ataxia, dysarthria, muscle weakness, manic depression and other psychotic episodes, and severe cerebellar atrophy (Solovyeva et al., 2018; Leal et al., 2020; Masingue et al., 2020). Adult chronic TSD patients have a less severe form of TSD and usually have residual enzymatic function (5–20% of normal activity) (Solovyeva et al., 2018). Patients can survive to geriatric age with proper symptom management (Neudorfer et al., 2005).

## Strategies for TSD Therapies

### Enzyme Replacement Therapy

Enzyme replacement therapy (ERT) has been successful for multiple lysosomal disorders including Gaucher, Fabry, Mucopolysaccharidosis type (MPS) I, MPS II, MPS IVA, MPS VI, MPS VII, and Pompe diseases (Barton et al., 1991; Eng et al., 2001; Klinge et al., 2005; Concolino et al., 2018). The first ERT for Gaucher disease was approved by the U.S. Food and Drug Administration (FDA) in 1991 using the glucocerebrosidase (GC) enzymes purified from human placenta (Ries, 2017) and was later replaced by imiglucerase, a recombinant human protein that is now broadly used in ERT. Currently, the field uses recombinant human enzymes for all ERT. The recombinant proteins are administered intravenously, and enter lysosomes through endocytosis, where the enzymes exhibit their functions to ameliorate disease phenotype in patients. However, it has also been found that the therapeutic efficacy may vary from patient to patient; and not all tissues and organs respond to ERT equally (Xu et al., 2016). Specifically, recombinant proteins cannot penetrate the blood brain barrier (BBB) and thus have no therapeutic effects on neuronal symptoms of diseases. In addition, the half-life of recombinant proteins is short which necessitates repeated intravenous administration of large amounts of recombinant proteins. ERT is generally very expensive and is often financially burdensome for patients, their families, and society (Rombach et al., 2013; Dussen et al., 2014). Development of immune response and generation of neutralizing antibodies to ERT is a major issue during treatment that often leads to treatment discontinuation. Clinical detections of immune response and antibodies to ERT, as well as mechanistic studies on immune response generation are still needed (Kishnani et al., 2016). Given the dearth of other effective treatments for LSDs, ERT is still an important therapeutic tool for the LSD community. Countermeasures of immune response to ERT may be considered for further study and development. To facilitate BBB penetration of ERTs, nanoparticle formulation of GC proteins is being studied to treat the neuronal symptoms of Gaucher disease (Sun et al., 2020). However, while promising, this approach is still in the preclinical stage. Direct CNS administrations of ERT have also been tested in a few LSDs via intrathecal injection for Hurler syndrome (Akeboshi et al., 2007) and MPS I (Concolino et al., 2018; Chen et al., 2020), and intraventricular infusion for neuronal ceroid lipofuscinosis type 2 (CLN2) (Schulz et al., 2018). The long term clinical therapeutical benefits of the ERT through CNS administrations are still under investigation (Marchetti et al., 2022).

For TSD, ERT remains challenging because a successful ERT must comprise of both a functional alpha and beta subunit of HexA. Several groups have developed functional HexA enzyme which have alleviated disease phenotypes *in vitro* (Akeboshi et al., 2007; Tsuji et al., 2011). Due to the large size of HexA, it is unable to cross the blood brain barrier and therefore traditional intravenous administration has not been effective in alleviating central nervous system symptoms (Leal et al., 2020). To overcome this challenge, alternative routes of HexA administration have been attempted. Intracerebroventricular (ICV) injections of a modified HexB, with both HexA and HexB activities, into Sandhoff’s disease mouse models showed marked reductions in GM2 and a two-fold increase in Hex activity, as well as an observed reduction of GM2 in the liver (Matsuoka et al., 2011). The modified HexB could potentially be developed as a common treatment for both TSD and SD but would not treat GM2 AB variant patients. ICV administration has not been attempted on TSD patients given that it is not a convenient drug administration route.

### Enzyme Enhancing Therapy: Pharmacological Chaperones

Pharmacological chaperones are small molecule drugs that bind to mutant proteins after their synthesis to correct their conformation such that the mutant proteins can be transported correctly to their cellular site for their biological functions. In many LSDs with missense mutations, mutant enzyme proteins are synthesized but cannot be trafficked to lysosomes due to misfolding and ER-retention, and could potentially be targeted by pharmacological chaperone therapy (Xu et al., 2016). The advantages of small molecule chaperone therapy include potentials for convenient oral administration, the capability to penetrate the BBB to treat neuronal symptoms, lack of immunogenicity issues (compared with ERT), and lower manufacturing costs. A common, but counterintuitive, method to identify chaperone compounds is to screen for enzyme inhibitors because the inhibitors could bind tightly to enzymes and stabilize their tertiary structure to promote folding and trafficking of mutant enzymes out of the ER and to lysosomes (Xu et al., 2016). Using this strategy, migalastat was identified as a small molecule inhibitor of alpha-galactosidase (alpha-GalA) with chaperone properties (Sanchez-Fernandez et al., 2016), and became the first in class pharmacological chaperone to be approved by the FDA for treatment of adults with Fabry disease (Administration, 2018b; Germain et al., 2019). Migalastat is indicated for a subset of Fabry patients with amenable mutations in alpha-GalA (Administration, 2018a). Similarly, ambroxol was identified as a chaperone for a subset of GC mutants (Maegawa et al., 2009b), and was tested in clinical trials for Gaucher disease (Zimran et al., 2013; Narita et al., 2016). Ambroxol is approved in most countries, but not the US, as an expectorant and shows promise to be repurposed for treatment of Gaucher disease. Initial small scale open-label clinical trials found that oral ambroxol was well tolerated in Gaucher patients, crossed the BBB, increased GC activity in lymphocytes, and ameliorated disease phenotypes in some patients (Zimran et al., 2013; Narita et al., 2016).

For TSD, some mutations cause misfolded HexA enzymes that are degraded quickly before the enzyme can move to the lysosome. One potential solution for these misfolded HexA enzymes is the use of pharmacological chaperones to promote proper folding in patient cells. One chaperone compound, pyrimethamine (PYR), has been found to inhibit HexA degradation long enough for the enzyme to reach the lysosome and enact on GM2 hydrolysis *in vitro* (Maegawa et al., 2007). Specifically, PYR binds to the active site of domain II in HexA which stabilized the enzyme in cells carrying the late-onset mutation alphaG269S (Maegawa et al., 2007). PYR is an approved therapy for toxoplasmosis and was repurposed in several clinical trials to evaluate its effects in TSD patients. However, during these trials, the patients only showed temporary increases in HEXA activity, which did not lead to improvements in symptoms, and was accompanied by undesirable side effects (Clarke et al., 2011; Osher et al., 2015).

More recently, small molecule binders of enzymes without inhibitory activity that can act as chaperones have been proposed and studied (Han et al., 2020; Liguori et al., 2020). These new types of chaperone compounds should have better efficacy to increase functions of mutant enzymes than the inhibitor chaperone compounds. PYR was identified from an *in vitro* screen of approved drugs (Maegawa et al., 2007). It is possible that a larger scale screen might identify more promising chaperones against HexA.

### Substrate Reduction Therapy

Substrate reduction therapy (SRT) is a therapeutic strategy to inhibit the formation of specific substrates of a mutant enzyme that reduces the need of this enzyme to hydrolyze its substrate, resulting in a decrease in the substrate accumulation of the lysosomal storage disease. For example, miglustat (N-butyldeoxynojirimycin, NB-DNJ), is an immunosugar inhibitor of glucosylceramide synthase that reduces the substrate accumulation due to the mutations of glucocerebrosidase in Gaucher disease (Pastores et al., 2009). For TSD, SRT involves inhibiting the production of the accumulating substrate, e.g. reducing GM2 produced in TSD. The small molecule, miglustat, was tested in animal models and shown to be partially efficacious. It was found to reduce GM2 in TSD mouse model brains by 50% via competitive inhibition of glucosylceramide synthase (Bembi et al., 2006; Solovyeva et al., 2018). It was then tested in multiple clinical trials with little success. Miglustat was tested for clinical efficacy in two infantile patients due to promising attributes such as its ability to pass through the BBB as well as success in other diseases (Platt and Jeyakumar, 2008; Leal et al., 2020). However, it did not prevent neurological symptoms from progressing (Bembi et al., 2006). Miglustat was also assessed in five patients with the juvenile form of GM2 gangliosidosis over the course of 2 years without showing improvement to neurological signs and symptoms (Maegawa et al., 2009a). Furthermore, adults with SD underwent a 3-year treatment of miglustat and were tested for efficacy towards alleviating neurodegeneration without success (Tavasoli et al., 2018; Leal et al., 2020). While miglustat did not clinically benefit the neurological impairment of these chronic GM2 gangliosidoses patients, the question remains about the early intervention of miglustat or other SRTs. Implementation should occur in the early stages of disease progression to see if the SRT has the ability to prevent neurodegenerative signs and symptoms. To date, miglustat is not approved by the U.S. Food and Drug Administration for TSD or SD.

Another SRT that has been investigated is Genz-529468 due to its IC_50_ that is 250-fold more potent than miglustat (Ashe et al., 2011). Genz-529468 works as a glucosylceramide synthase inhibitor similar to miglustat, though its full mechanism of action has not been described (Ashe et al., 2011). Mice treated with Genz-529468 showed a delayed loss of motor function and longer lifespan despite an increase in intracellular GM2. Furthermore, mice treated with both miglustat and Gen-529468 showed anti-inflammatory responses such as lowered microglial activation, lowered astrogliosis, and delayed neuronal apoptosis, which indicates the use of anti-inflammatory SRTs that can pass through the BBB could serve as potential therapies for GM2 gangliosidosis disorders (Ashe et al., 2011).

### Hematopoietic Stem Cell Transplantation

HSCT is mainly achieved through transplantation of stem cells from peripheral blood, bone marrow, or umbilical cord blood (Solovyeva et al., 2018; Leal et al., 2020). This is possible because Hex enzymes are able to pass from cell to cell through the M6PR-mediated process (Leal et al., 2020). HSCT has been successful in several LSDs including MPS I (Visigalli et al., 2010), MPS II (Gleitz et al., 2018), and Gaucher disease type 1 and 2 (Somaraju and Tadepalli, 2017). Bone marrow transplantation in SD mice showed an increase in survival rate in one study (Norflus et al., 1998) and prevention of neurological degeneration via decreased microglial activation in another study (Wada et al., 2000). One effort by Jacobs, et al. treated a 3-year old TSD presymptomatic patient with a bone marrow transplant. Results showed increased HexA activity in leukocytes and plasma but no prevention of neurological signs and symptoms (Jacobs et al., 2005). Another effort in which a bone marrow transplant from a healthy donor matched sibling to a late-onset 15 year-old TSD patient resulted in HexA activity comparable to normal (control group) levels in leukocytes and other somatic tissue 8 years after the completed graft (Stepien et al., 2018). While the HSCT did not alleviate the patient’s signs and symptoms, it did prevent neurological degeneration such that his signs and symptoms were “tolerable for daily life” (Stepien et al., 2018).

While this effort showed the promise of HSCT, major hurdles include the challenge of finding a proper donor match and negative immunogenic responses of a less than perfect match. Recent efforts have focused on taking healthy donor stem cells or a patient’s own gene therapy corrected stem cells; and transplanting them into a patient (Leal et al., 2020). Immune response issues have been problematic when using healthy donor control stem cells, however. The patient autologous, gene therapy corrected stem cell replacement therapy is promising, and currently being tested in the clinics for several LSDs but has not yet been tested in TSD patients (Nagree et al., 2019).

### Gene Therapy

Gene therapy utilizes viral vectors for the delivery of a functional gene to correct a genetic defect. Since TSD defects are monogenic, gene therapy is a promising treatment for this disease. Gene therapy administration could be *ex vivo*, where patient autologous cells are isolated, transduced with viral vectors, and reintroduced into the body, or *in vivo*, where the gene therapy is directly administered to the patient. The predominant viral vector used for *in vivo* gene therapy is the adeno-associated virus (AAV), whereas *ex vivo* vectors are predominantly retroviral, such as HIV-1-derived lentivirus or gamma-retrovirus vectors. AAV vectors have been administered in an estimated 250–300 clinical trials and have had a good overall safety record to date in human patients (Wang et al., 2019). Currently, several gene therapies have been approved by the FDA and European Medicines Agency (EMA), one of which is for an LSD. Libmeldy is approved by the EMA as an *ex vivo* gene therapy treatment for metachromatic leukodystrophy (MLD) and is currently in clinical trials in the US (Fumagalli et al., 2022). The *ex vivo* gene therapy strategy for LSDs is to transduce hematopoietic stem cells isolated from the patient with the gene of interest as a modified hematopoietic stem cell transfer (HSCT) therapy, with the advantage of using autologous cells. Gene therapies have been tested in several clinical trials for LSDs, both *ex vivo* (Dunbar et al., 1998; Khan et al., 2021) and *in vivo* (Worgall et al., 2008; Smith et al., 2013; Tardieu et al., 2014; Tardieu et al., 2017). For *in vivo* gene therapy, since AAVs do not cross the blood brain barrier, several of the trials for LSDs with neurological symptoms were conducted using local administration via intracerebral or intrathecal routes, and the gene therapies were found to be well tolerated (Worgall et al., 2008; Tardieu et al., 2014; Tardieu et al., 2017). This is promising for TSD due to its predominantly neurological nature. Gene therapy is an active area of development for LSDs, with much preclinical activity and many ongoing clinical trials (Nagree et al., 2019).

The unique challenge of gene therapy for TSD is the fact that *HEXA* encodes one subunit of the heterodimer HexA enzyme, and needs to complex with the beta subunit, encoded by *HEXB*, in order to be functionally active. Therefore, gene therapy development for TSD requires the delivery of two subunits to form a functional enzyme. However, the advantage of this strategy is that a single therapy could be used to treat two GM2 gangliosidosis diseases, TSD and SD, although not the AB variant disease. In order to do so, several strategies have been tested in animal models including injection of a 1:1 mix of monocistronic AAV containing *HEXA* and *HEXB*, delivery of a hybrid beta-hexosaminidase subunit (*HEXM*) capable of forming stable homodimers, and use of bicistronic AAV vectors to deliver both *HEXA* and *HEXB* (Bradbury et al., 2015; Gray-Edwards et al., 2018; Lahey et al., 2020; Taghian et al., 2020). AAV-based therapies have been successful in reducing GM2 in mice and cats but failed to reduce GM2 in the central nervous system of non-human primates (Osmon et al., 2016; Golebiowski et al., 2017; Leal et al., 2020). Recently, the bicistronic AAV gene therapy approach is the first to reach the clinics with a phase 1/2 registered trial (NCT04798235) that began in 2021. This trial will evaluate safety and efficacy of TSHA101, a HEXBP2a-HEXA transgene packaged in AAV9 and administered via intrathecal injection to TSD and SD patients (Sehgal, 2021). Recent efforts are investigating the use of CRISPR/Cas9 for targeted correction of the mutated *HEXA* gene, but these efforts are still in the early stages of development (Tropak et al., 2016). However, a limitation of AAV gene therapy is immunogenic responses against the AAV capsid, which might limit administration to a single dose, and will need to be monitored in the clinics and in preclinical animal studies (Rabinowitz et al., 2019). Re-administration of AAV therapy is currently being evaluated in a clinical trial for Pompe disease (NCT02240407).

### Therapies With mRNA and Antisense Oligonucleotide

Recent developments in antisense oligonucleotide (ASO) technology provides additional therapeutic development strategy. Briefly, ASOs utilize a single-stranded oligonucleotide that binds to ribonucleic acids to modify gene expression or gene splicing, and therefore enzymatic expression and activity (Crooke et al., 2021). Currently, nine ASO drugs have been approved by the FDA and the EMA (Crooke et al., 2021). The predominant mechanisms of action of the approved ASOs are knockdown of target genes, and correction of frameshift mutations through exon skipping. However, nusinersen, which is approved for spinal muscular atrophy (SMA), works through a different mechanism. Nusinersen corrects splicing of a redundant gene, survival motor neuron 2 (*SMN2*), to compensate for loss of SMN1 function, by increasing exon 7 inclusion. Without nusinersen, splicing of SMN2 mRNA excludes exon 7 and produces a shortened, nonfunctional protein, which is quickly degraded (Edinoff et al., 2021). Efficient delivery of ASOs to target cells remains a challenge *in vivo*, but the approval of nusinersen for SMA, which is administered via intrathecal injection, provides precedence for direct CNS delivery (Edinoff et al., 2021).

ASO development for LSDs is still in the proof of principle stage. ASOs have been shown to correct the most common mutation in late onset Pompe disease, a T-to-G mutation in intron 1 of the alpha-glucosidase gene, which causes an exon 2 exclusion splicing error. The ASOs were directed towards splicing silencer sequences within either exon 2 or intron 1, and increased exon 2 inclusion in Pompe patient-derived fibroblasts and myotubes (Goina et al., 2017; van der Wal et al., 2017). In Niemann-Pick type C (NPC) patient fibroblasts, ASO treatment was successful in blocking a cryptic splice site created by a G-to-A mutation in intron 9 of the NPC1 gene to correct mutant pre-mRNA splicing (Rodriguez-Pascau et al., 2009). Another approach is to use ASOs for genetic SRT. Clayton, *et al.* used ASOs to down regulate glycogen synthase 1 (Gys1) in Pompe disease mice and showed successful reduction in Gys1 activity and muscle glycogen levels (Clayton et al., 2014).

ASO development has not been explored for TSD. Because HexA is a compact enzyme with multiple regions throughout the protein that are critical to form the catalytic pocket, dimerization surface, GM2 activator binding site, disulfide bonds, and post-translational modifications necessary for trafficking, the exon skipping strategy is unlikely to be successful (Ohno et al., 2008). It is possible that the genetic SRT approach with ASOs might be useful for TSD. Additionally, of the over 200 known *HEXA* mutations along chromosome 15q23, 43 (∼19%) cause splicing defects (Cooper et al., 2020). It stands to be experimentally determined which of these would be amenable to ASO correction. Most of these mutations affect small patient numbers, therefore, drug development would need to follow the individualized medicine, or *n* = 1, approach that is currently being explored in other rare diseases, such as the case of milasen. Milasen is an ASO that corrects mis-splicing caused by a retrotransposon insertion in intron 6 of *CLN7*, a Batten disease causative gene. It was developed to treat a unique genetic mutation in a single patient; and went from whole genome sequencing diagnosis to FDA approval and patient dosing in less than a year (Kim et al., 2019).

## *In Vitro* and *in Vivo* Models of TSD for Therapeutics Development

### Model Development Considerations

**FIGURE 2:**
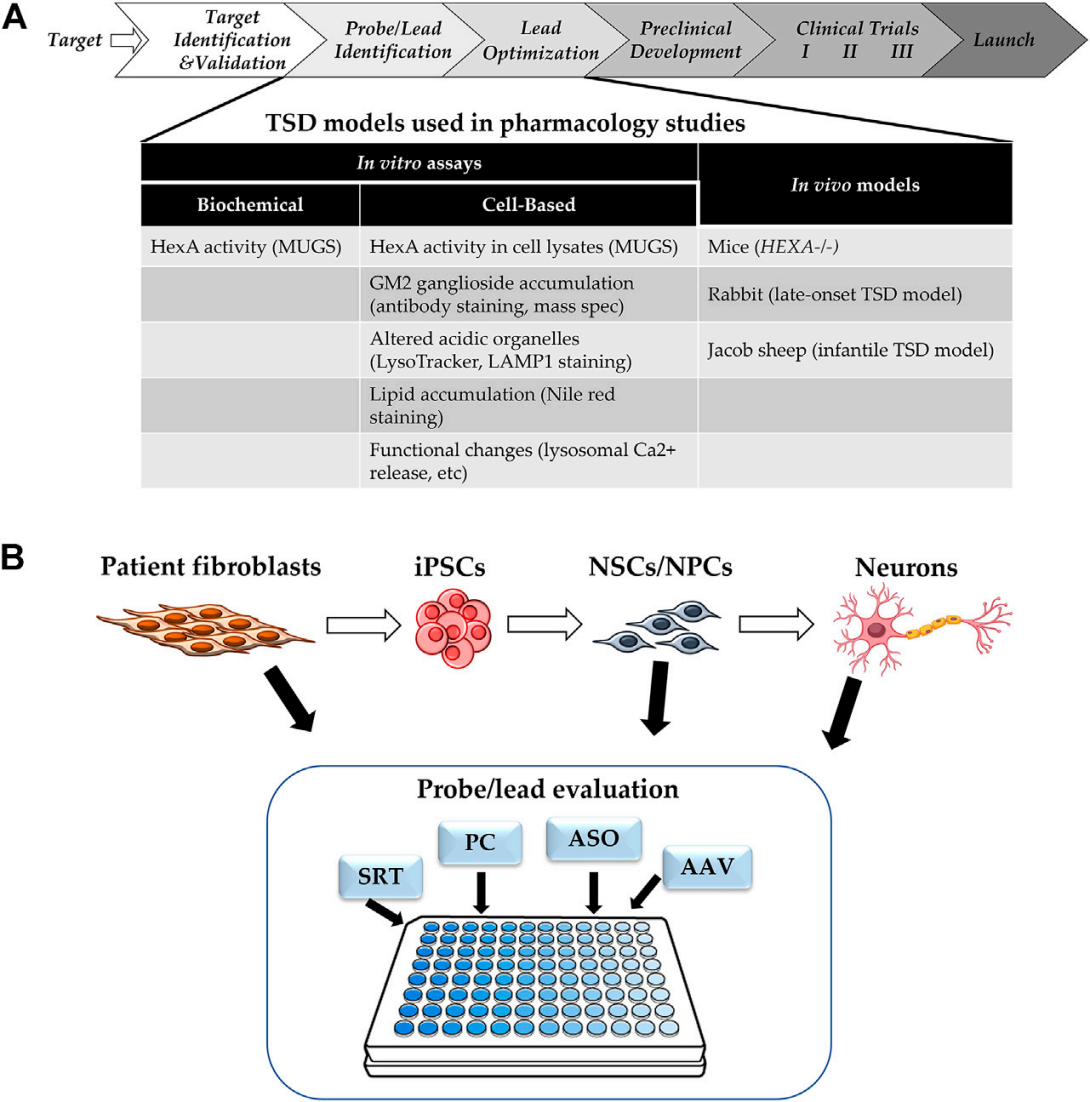
**(A)** Drug Development Progression. Pipeline of drug development with TSD specific assays and models. **(B)** Cell-based Assays for TSD. SRT: substrate reduction therapy, PC: pharmacological chaperone, ASO: antisense oligonucleotide, AAV: adeno-associated virus for gene therapy.

The typical drug development pipeline starts with target identification and validation, then moves on to hit and lead identification, lead optimization, preclinical development with investigational new drug (IND)-enabling studies, before reaching phase I clinical trials (Figure 2A) (Frederickson, 2012). Regardless of the type of therapeutic modality being developed, robust and reproducible *in vitro* assays and *in vivo* models that are representative of human diseases are critical drivers of the probe/lead identification and lead optimization stages. *In vitro* assays for TSD fall into two categories: HexA enzyme activity assays, and phenotypic assays. The consideration for assay readout depends on the mechanism of action of the therapeutics tested. Therapeutic modalities aimed at correcting HexA enzyme production, trafficking, and/or activity could be evaluated by either HexA enzyme activity assays in cell lysates (Tropak et al., 2010) or downstream phenotypic correction assays. Modalities targeting compensatory pathways, such as SRT, require assays for phenotypic corrections in TSD cells. The type of cells used for *in vitro* modeling must show decreased HexA activity and cell-based TSD phenotypes.

Another consideration is assay throughput. The goal of the probe/lead identification and the lead optimization stages is to narrow down the list of candidates to a single lead to enter the resource intensive preclinical development stage. The number of candidates evaluated depends on the type of therapeutic modalities. For small molecules, a typical high throughput screen could start with >100,000 compounds and require a large number of cells, as well as assays that are amenable to be run at scale. Alternatively, a pharmacological chaperone screen for HexA tested an FDA-approved drug collection of ∼1,000 compounds (Maegawa et al., 2007), which would require medium throughput assays. Large molecule and biologics modalities typically start with fewer candidates for evaluation. Candidates are prioritized and narrowed down to a manageable number for further testing in *vivo* models.

### HexA Enzyme Activity Assays

Synthetic fluorogenic substrates can be used to assay Hex activity from purified enzymes or cell lysates. Total Hex activity can be measured using 4-methylumbelliferyl-β-N-acetylglucosamine (MUG), whereas, 4-methylumbelliferyl-β-N-acetylglucosamine-6-sulfate (MUGS) is used to measure HexA specific activity. The biochemical MUGS assay has been used for high throughput screens of compound libraries for pharmacological chaperones, and to evaluate activity of HexA enzymes intended for ERT (Akeboshi et al., 2007; Maegawa et al., 2007). The MUGS assay is part of the clinical diagnostic tests for TSD patient, using a variety of patient samples such as patient cells, biological fluids, or dried blood spots (Hall et al., 2014). One type of patient cells used for clinical diagnosis is dermal fibroblasts, which can be isolated with minimally invasive procedures, expanded for up to 20 passages in 2D culture, and immortalized with human telomerase reverse transcriptase (hTERT) transfection if a large number of cells is needed (Funk et al., 2000). TSD patient-derived fibroblasts have been cultured *in vitro* and used for drug efficacy evaluations with HexA activity assays (Tropak et al., 2004; Tropak et al., 2016).

### Phenotypic Cell-Based Assays

Cell-based models of TSD show phenotypes such as GM2 ganglioside accumulation, as well as downstream phenotypes caused by this accumulation, such as increased staining of acidic organelles by LysoTracker or LAMP1, accumulation of lysosomal lipids stained by nile red, and/or altered lysosomal calcium release (Xu et al., 2014; Vu et al., 2018; Colussi and Jacobson, 2019; Matsushita et al., 2019). Most of the cell-based assays for TSD use patient-derived fibroblasts, but recently, there has been more research using patient induced pluripotent stem cells (iPSC) and derived neuronal cells, as the majority of the patient phenotypes is CNS-related (Liu and Zhao, 2016; Vu et al., 2018; Matsushita et al., 2019) (Figure 2B). Furthermore, TSD patient-derived neuroprogenitor cells have been differentiated into neurons and showed presynaptic defects in FM1-43 dye uptake (Matsushita et al., 2019).

Another type of phenotypic assay that directly measures GM2 ganglioside accumulation in cells is that using mass spectrometry. Some major benefits for using mass spectrometry (MS) are the speed, low sensitivity and detection limits, lack of need for coupling agents or labels, and amenability to a wide variety of molecular classes. Furthermore, MS enables detection of analytes within a complex sample that is often not able to be analyzed via other techniques (Roddy et al., 2007). This is possible because MS technology ionizes the entire contents of a sample and outputs the analysis of an experiment based on mass alone (if using a standalone mass spectrometer). Two main types of MS have evolved for phenotypic assays: liquid chromatography—mass spectrometry (LC-MS or LC-MS/MS) and matrix assisted laser desorption ionization—mass spectrometry (MALDI-MS) (Haslam et al., 2016; Rohman and Wingfield, 2016). LC-MS uses electrospray ionization in order to generate ionized aerosol droplets of a sample whereas MALDI-MS irradiates a matrix-laden sample using a laser to generate a plume of ionized gas-phase molecules. Both technologies are good candidates for performing phenotypic assays for LSDs. Specifically, both MS technologies can be used to identify proteomic and metabolomic targets for various LSD therapies (Parenti et al., 2021). There is a history of using MS technology to identify biomarkers for LSDs including Fabry disease, Niemann-Pick disease Type C, MSP, and oligosaccharidosis (Mashima et al., 2020). However, there is little evidence of performing a phenotypic screen for an LSD using MS technology.

### *In Vivo* Models

The available *in vivo* models of TSD include mice, rabbits, and sheep. The first murine model of TSD was developed in 1995 by Taniike et al. in which a knockout of *HEXA* was performed. While these mice had deficient HexA activity, they did not present classical TSD phenotypes. For example, GM2 accumulated in only a few regions of the brain including the olfactory bulb, cerebral cortex, and the anterior horn of the brain (Taniike et al., 1995). Additionally, these mice had a normal lifespan. Differences in phenotype and physiology of these *HEXA* ^−/−^ mice can be attributed to the fact that ganglioside metabolism between humans and mice is not the same. Mice have additional redundant sialidases that humans do not have that can enact on GM2 (Solovyeva et al., 2018).

Qian, et al. have used prime editing (PE) to develop a TSD rabbit model. Briefly, PE is a “search-and-replace” genome editing technology that doesn’t rely on double-strand breaks or donor DNA (Anzalone et al., 2019). Additionally, PE can perform all 12 possible point-mutation changes whereas other technologies have been resigned to eight point-mutations (Anzalone et al., 2019). To develop the rabbit model, a four-base insertion of TATC in exon 11 of *HEXA* was performed to mimic the TATC insertion mutation present in 80% of patients with TSD in the Ashkenazi Jewish population (Frisch et al., 2004). Enzymatically, this TATC insertion prematurely terminates translation of *HEXA* and leads to decreased activity of HexA. Phenotypically, the rabbits presented manifestations similar to that of late-onset TSD patients such as ataxic gait, convulsions, muscle fibrosis, and enlargement of the perineural space (Anzalone et al., 2019).

Another *in vivo* model for TSD is Jacob sheep, which carries a naturally occurring *HEXA* mutation. Four sheep, aged 6–8 months, exhibited signs of a progressive neurodegenerative disorder. Analysis of lysosomal enzymes in brain and liver tissue from these animals indicated reduced HexA activity as well as an accumulation of GM2. Genomic analysis revealed a missense mutation within exon 11 of the sheep’s cDNA (Torres et al., 2010). Affected Jacob sheep had phenotypic signs similar to those in human infantile TSD such as ataxia, proprioceptive deficits, cortical blindness, astrocytosis, and microgliosis (Torres et al., 2010; Porter et al., 2011). Jacob sheep with TSD present an *in vivo* system that most closely mimics that of human TSD. However, no therapies have been tested in TSD Jacob sheep yet. The downside of a large animal disease model is that more of the drug candidate will need to be manufactured for *in vivo* studies which could represent a significant financial hurdle.

## Perspectives and Conclusion

### Perspectives on *in Vitro* Assays

When considering *in vitro* assays to support drug discovery and development efforts, it is preferable, although not always possible, to measure the same enzyme activity or analyte in *vitro* cell-based assays, *in vivo* efficacy studies, and as a biomarker in the clinics. Since most of therapeutic strategies currently being explored for TSD aim to restore HexA enzyme function (ERT, pharmacological chaperone, gene therapy, and ASO), assays for HexA activity in patient cells could be used to triage hits to support development of these therapeutic modalities, and would also provide patient genetic context for ASO evaluations. Assaying HexA activity using the MUGS substrate is commonly performed on patient cells as a key part of TSD diagnosis (Hall et al., 2014). MUGS and MUG assays are also commonly used to measure HexA and total Hex activities in brain tissues of TSD and SD animal models for efficacy evaluations (Matsuoka et al., 2011; Osmon et al., 2016; Gray-Edwards et al., 2018; Lahey et al., 2020). HexA activity was also measured in TSD patients’ peripheral blood mononuclear cells (PBMCs) and plasma in two separate clinical trials to evaluate the pharmacological chaperone PYR treatment, and in CSF in an ongoing gene therapy trial (Clarke et al., 2011; Osher et al., 2015; Sehgal, 2021).

Another common assay for TSD and SD *in vivo* efficacy studies is evaluation of GM2 accumulation in brain tissue for TSD and SD models, and in liver tissue for SD models (Matsuoka et al., 2011; Osmon et al., 2016; Tropak et al., 2016; Gray-Edwards et al., 2018; Lahey et al., 2020). However, crossover to the clinics is limited, as GM2 accumulation in TSD patient brains could only analyzed in autopsies. Similarly, TSD-patient derived iPSCs differentiated into neural lineages also show the GM2 accumulation phenotype (Vu et al., 2018).

### Perspectives: HTS of Cellular TSD Model

**FIGURE 3:**
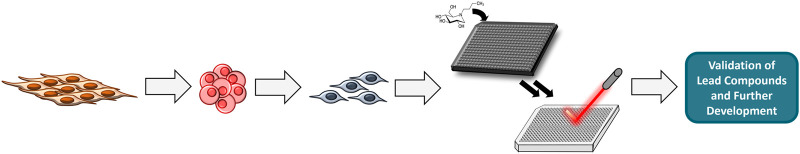
General workflow for performing HTS. Fibroblasts are reprogrammed into iPSCs which are programmed into NSCs. HTS is done using NSCs and MS.

One of the more difficult aspects of treating TSD is trying to ameliorate the central nervous system signs and symptoms. Using recent advances in stem cell technology, efforts have been made to study neural stem cells (NSCs) which model GM2 accumulation in central nervous system cells (Vu et al., 2018). These NSCs can then be used as a cell-based model system to evaluate drug efficacy and promote drug development. A general scheme for this workflow is shown in Figure 3. Specifically, dermal fibroblasts are taken from TSD patients and used to generate induced pluripotent stem cells (iPSCs) using a non-integrating Sendai virus reprogramming assay. Once generated, the iPSCs are grown until they are clear of the Sendai virus before being transformed into NSCs using commercially available assay kits (Vu et al., 2018). The NSCs are then stained with various lipid markers including nile red, Lysotracker red (LAMP1), filipin, and GM2. These markers are used as validation to insure the NSCs are presenting the characteristic GM2 accumulation of TSD. Once validated, high throughput screening for drug efficacy using mass spectrometry can be performed.

To enable HTS, NSCs can be grown in a high-density multi-well format (e.g., 384- and 1536-well plates). Compound collections of choice are then tested. In order to analyze if these compounds have effectively reduced GM2 in a high-throughput label-free manner, mass-spectrometry can be used. Cells treated with compounds are lysed and then fortified with a heavy-labeled internal standard (e.g., 13C-GM2) which allows for quantification of GM2 when compared to controls. Using MALDI-MS, high throughput analysis of the cell lysates is possible. After analysis, any lead compounds will be validated and further developed for treatment of TSD.

## Conclusion

### Perspectives and Conclusion

Tay-Sachs disease is an autosomal recessive disease caused by *HEXA* mutations. It is mainly characterized by neurodegenerative clinical manifestations in patients. While there is a wide gradient of signs and symptoms, the age of onset generally indicates disease severity and patient outcome. While there exist some therapies for symptom management, especially for late onset TSD patients, currently, there is no cure for TSD. Several approaches have been reported to develop therapies for TSD including enzyme replacement therapy, enzyme enhancement therapy via the use of chaperones, substrate reduction therapy, gene therapy, and stem cell replacement therapy. In order to test developed therapies, *in vitro* and *in vivo* TSD models exist. This includes HexA enzymatic assays (e.g. MUGS) and phenotypic assays like that using MS technology for cell-based systems; and the use of mice, rabbits, and sheep for *in vivo* systems. This review suggests a novel methodology for discovering small molecules for substrate reduction and pharmacological chaperone therapies. Specifically, we propose to use MALDI-MS to perform HTS of compound libraries. This methodology enables direct quantitation of GM2 without labels of dyes. The therapeutic strategies described herein as well as the ways to assess them via *in vitro and in vivo* experiments could result in lead therapies to be further developed for the treatment of TSD. While this review focused on research and therapeutic development for TSD, the importance of collaborations with patient advocacy groups needs to be mentioned, as such partnerships have had proven successes in bringing together scientists and patients to facilitate research and patient recruitment for clinical trials {Walkley, 2016 #128}.
